# A Systematic Review of Native Languages Mapped With Direct Electrical Stimulation During Awake Craniotomy

**DOI:** 10.7759/cureus.105487

**Published:** 2026-03-19

**Authors:** Salih Al-Ani, Hana Hallak, Aimun A Jamjoom

**Affiliations:** 1 Trauma and Orthopaedics, Torbay Hospital, Torquay, GBR; 2 Postgraduate Medical Sciences, University of Birmingham, Birmingham, GBR; 3 Neurological Surgery, Alfaisal University College of Medicine, Riyadh, SAU; 4 Neurological Surgery, Blizard Institute, Queen Mary University of London, London, GBR; 5 Neurosurgery, Queen's Hospital, London, GBR

**Keywords:** awake craniotomy, direct electrical stimulation, functional mapping, glioma, language research

## Abstract

Human language exhibits both universal characteristics and remarkable diversity. While neuroimaging has identified a shared fronto-temporo-parietal network across languages, causal inferences require techniques such as direct electrical stimulation (DES), which is performed during awake craniotomy. However, it is unclear which native languages have been mapped using DES. We conducted an analysis of which native languages were mapped in monolingual patients undergoing DES during awake craniotomy. Data was extracted from an open-access systematic review dataset of language errors from patients during awake craniotomy. An additional literature review and author communication were performed to identify the language type. Descriptive statistics and chi-squared analysis were used. The analysis identified 893 stimulation sites across 73 studies, involving nine languages: French, English, Chinese, Japanese, Italian, Dutch, Spanish, German, and Persian. These languages represent 27.2% of native languages spoken globally. The predominant mapped language family was Indo-European (670 stimulation sites; 75%), followed by Sino-Tibetan (186 stimulation sites; 20.8%) and Japonic (37 stimulation sites; 4.1%). There was a significant difference between the frequencies of the total number of global native speakers and stimulation sites (x^2^ = 2071.33, *df* = 28, p <0.00001). We calculated a ‘stimulation site per million native speakers’ metric and identified that the top mapped languages per population size were French (4.62), Dutch (0.99), and English (0.65). This study underscores a significant bias in DES-based language mapping literature, with under-representation of languages spoken by most of the global population. Standardized, multi-language testing paradigms are crucial for addressing these disparities and advancing inclusive cognitive neuroscience.

## Introduction and background

Human language is a complex system of communication that lies at the heart of building relationships, personal growth, and cultural dissemination. Globally, there is a huge diversity of human language with approximately 7000 spoken languages from over 100 language families [1]. Human languages are characterised by a set of universal characteristics that set them apart from other forms of animal communication. Charles Hockett defined 13 universal features of human language, including displacement (the capability of language to communicate about things that are not immediately present), cultural transmission (language is passed through socio-cultural interaction rather than genetics), and duality (use of a small number of meaningless sounds to produce a large number of meaningful words) [2]. Despite these universal features, human languages do differ in their phonology (tonal versus non-tonal languages), syntax, and writing systems. This raises the question of whether the neurobiology of language is based on a universal neural architecture or whether differing brain regions are involved with different languages.

Large-scale functional MRI (fMRI) studies have shown a common fronto-temporo-parietal network across 45 languages from 12 language families [3]. However, fMRI is an associative imaging modality and does not allow for the causal inference of function to brain anatomy. Several key criteria for defining causality in human brain mapping involve stimulation [4]. Neurosurgeons are in a unique position to study brain function during awake craniotomy with language mapping using direct electrical stimulation (DES). During awake craniotomy, neurosurgeons use DES to identify language errors by applying a brief electrical current to discrete cortical and subcortical regions while the patient performs naming or counting tasks, thereby identifying language-essential sites that must be preserved during tumour resection. The collection of this data has permitted the building of functional language maps using DES [5,6]. These maps have played a major role in shaping theoretical frameworks on the perception and production of language [7].

However, comparison of different languages mapped using DES revealed differences in the neuroanatomy of certain language errors [8]. This raises the question of which languages mapped with DES have been described in the literature. In particular, whether there is evidence of bias towards a small number of languages. To address this question, we conducted a secondary analysis of an open systematic review dataset to understand which native languages have been mapped using DES in awake craniotomy and how this relates to global native-speaker populations.

## Review

Methods

In this analysis, we asked the question: which native languages have been mapped in monolingual patients using DES during awake craniotomy? To answer this, an open-access dataset was leveraged from a previously published systematic review [9]. This review was performed according to the PRISMA statement guidelines and was registered on PROSPERO (CRD42020196727) [10]. The authors of the systematic review conducted a search using Embase, Medline Ovid, Web of Science, Cochrane, and Google Scholar for all articles published until 6th July 2020. The inclusion criteria for the studies were: all articles that reported on adult monolingual patients with gliomas (WHO grade II-IV) who underwent awake craniotomies with DES and had documented intraoperative language errors. Monolingual patients were selected to isolate native language effects on brain mapping without confounding multilingual influences. The systematic review included 102 studies with a total of 930 errors. First, we conducted a comprehensive assessment of the dataset to check its integrity, in particular examining for duplications (using author and year as a comparator). This was followed by a review of the references and our knowledge of the literature to determine if any additional studies could be included.

We attempted to contact the corresponding author of the systematic review if we identified any discrepancies in the dataset or identified non-included research articles that we thought met the inclusion criteria. Subsequently, we aimed to identify which language (ie, English, French, Chinese, etc) was mapped in the identified studies. To do this, we took a sequential approach, first examining the abstract and then the full research article. A language was included when explicitly stated in the article or abstract. This process was performed by two authors (SA, AJ), and any disagreements were discussed and resolved with the support of a third reviewer (HH). If the language was not found in either of these sources, we contacted the corresponding author. For a study to be in the final dataset, there needed to be confirmation of the type of language that was mapped from either the article or the corresponding author. Studies were excluded if the mapped language could not be identified from these sources or if the study included a mixed language population. The risk of bias was assessed using the Joanna Briggs Institute (JBI) Critical Appraisal Checklist for Cases Series [11]. 

Statistical analysis

For each language, *Ethnologue: Languages of the World, 16th Edition* was used to define which family the language belonged to and how many native speakers there were globally [1]. The data were published on the Center for Open Science (OSF) registries as an open-access dataset [12]. Descriptive analysis was used to define the frequency of the languages, language families, and intraoperative paraphasias. A chi-square goodness-of-fit test was used to compare the observed distribution of DES stimulation sites across the 30 most widely spoken languages globally (by native speaker count, sourced from *Ethnologue*) against the distribution expected if stimulation sites were proportional to native speaker populations. Expected frequencies were calculated by multiplying the total number of stimulation sites by each language's proportion of the combined native speaker population across all 30 languages. Our null hypothesis was that there was no difference between the native speaking population and DE stimulation sites in that language. Statistical significance was set at p < 0.05. A global heatmap of study numbers per country was generated using DataWrapper. Data visualisations were generated using R (R Foundation for Statistical Computing, Vienna, Austria) through the BioRender interface (BioRender, Toronto, Canada). 

Results

Study and Geographic Characteristics

**Figure 1:**
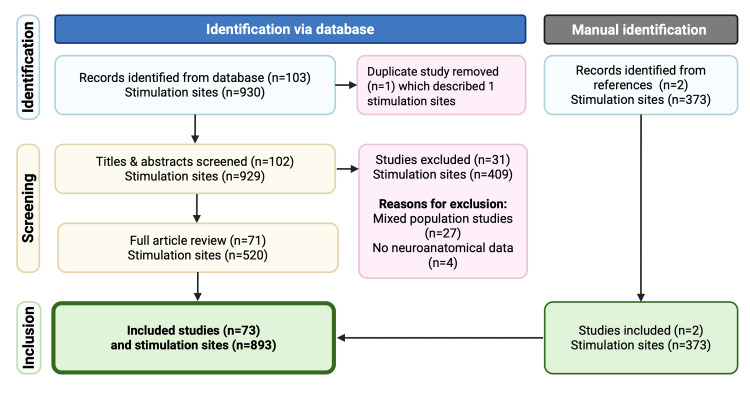
PRISMA flowchart PRISMA: Preferred Reporting Items for Systematic Reviews and Meta-Analyses

Initial review of the open-access dataset identified a duplicate study that described a single stimulation site. This yielded a dataset of 929 stimulation sites from 102 studies (Figure 1). We also identified two studies through citation chasing (a total of 373 stimulation sites) that we deemed to meet the inclusion criteria but were not in the initial systematic review [8,13]. We attempted to contact the corresponding author of the systematic review to ascertain why these studies were not included but got no response. After reviewing the articles and contacting corresponding authors, we excluded a total of 31 studies (there was no neuroanatomical data (n=4), and the author confirmed a mixed language population (n=27)). The final dataset for analysis included 893 stimulation sites from 73 studies [8,13-84] (Table 1). 

**Table 1:** Characteristics of included studies, including risk of bias

Study	Year	Language	Number of Stimulation Sites	Risk of Bias
Alimohamadi et al. [14]	2016	Persian	1	Moderate
Altieri et al. [15]	2019	Italian	2	Moderate
Barone et al. [16]	2018	Italian	2	Moderate
De Benedictis et al. [17]	2012	French	10	Moderate
De Benedictis et al. [18]	2010	French	13	Moderate
Benzagmout et al. [19]	2007	French	9	Moderate
Breshears et al. [20]	2019	English	2	Moderate
Bresson et al. [21]	2013	French	6	High
Chan-Seng et al. [22]	2014	French	39	Moderate
Chang et al. [23]	2011	English	11	Low
Chernoff et al. [24]	2019	English	6	High
Duffau et al. [25]	2001	French	5	High
Duffau et al. [27]	2009	French	12	Low
Duffau et al. [26]	2008	French	34	Moderate
Duffau et al. [28]	2009	French	7	Low
Fernández et al. [29]	2020	Spanish	14	Moderate
Fujii et al. [30]	2015	Japanese	7	Moderate
Gayoso et al. [31]	2019	Spanish	1	Moderate
Geemen et al. [32]	2014	French	21	Moderate
Gharabaghi et al. [33]	2006	German	1	High
Gil-Robles et al. [34]	2013	French	5	Moderate
Herbet et al. [35]	2017	French	4	Moderate
Hirono et al. [36]	2018	Japanese	1	High
Hiroshima et al. [37]	2014	Japanese	6	Moderate
Iijima et al. [52]	2017	Japanese	1	High
Joswig et al. [38]	2016	German	4	Moderate
Kamada et al. [39]	2007	Japanese	6	Moderate
Kemerdere et al. [40]	2016	French	12	Moderate
Khan et al. [41]	2014	French	5	Moderate
Kurimoto et al. [42]	2010	Japanese	1	High
Lang et al. [43]	2001	English	2	Moderate
Magrassi et al. [44]	2010	Italian	9	High
Maldonado et al. [45]	2011	French	30	Moderate
Martino et al. [46]	2012	Spanish	3	High
Matsuda et al. [47]	2014	French	38	Moderate
Moritz-Gasser et al. [48]	2013	French	10	High
Morrison et al. [49]	2016	English	5	Moderate
Morrison et al. [50]	2016	English	1	Moderate
Motomura et al. [51]	2014	Japanese	4	High
Motomura et al. [54]	2019	Japanese	3	Low
Motomura et al. [53]	2019	Japanese	3	Low
Mukae et al. [55]	2017	Japanese	4	Moderate
Nomura et al. [56]	2013	English	3	High
Oelschlägel et al. [57]	2020	German	1	Moderate
Ogawa et al. [58]	2014	Japanese	1	Moderate
Pallud et al. [59]	2019	French	2	High
Papagno et al. [60]	2011	Italian	5	Low
Parney et al. [61]	2010	English	1	High
Petrovich et al. [62]	2014	English	2	High
Petrovich et al. [63]	2005	English	5	Low
Plaza et al. [64]	2009	French	5	High
Pouratian et al. [65]	2004	English	12	High
Rech et al. [66]	2020	French	6	Moderate
Rech et al. [67]	2017	French	1	Moderate
Gil Robles et al. [68]	2005	French	10	Moderate
Rofes et al. [69]	2017	Italian	4	Moderate
Ruge et al. [70]	1999	English	5	Moderate
Sanai et al. [13]	2008	English	187	Low
Sarubbo et al. [71]	2011	Italian	2	Moderate
Satoer et al. [72]	2014	Dutch	1	High
Sierpowska et al. [73]	2015	Spanish	2	High
Southwell et al. [74]	2017	English	1	Low
Spena et al. [75]	2015	Italian	1	High
Tomasino et al. [76]	2015	Italian	5	High
Vassal et al. [77]	2014	French	3	Moderate
Vidorreta et al. [78]	2011	French	24	Moderate
Wilden et al. [79]	2013	English	1	Moderate
De Witte et al. [80]	2015	Dutch	11	Moderate
De Witte et al. [81]	2015	Dutch	13	Moderate
Wu et al. [8]	2015	Chinese	186	Low
Yordanova et al. [82]	2011	French	4	Moderate
Zammar et al. [83]	2018	English	2	High
Zemmoura et al. [84]	2015	French	27	Moderate

**Figure 2:**
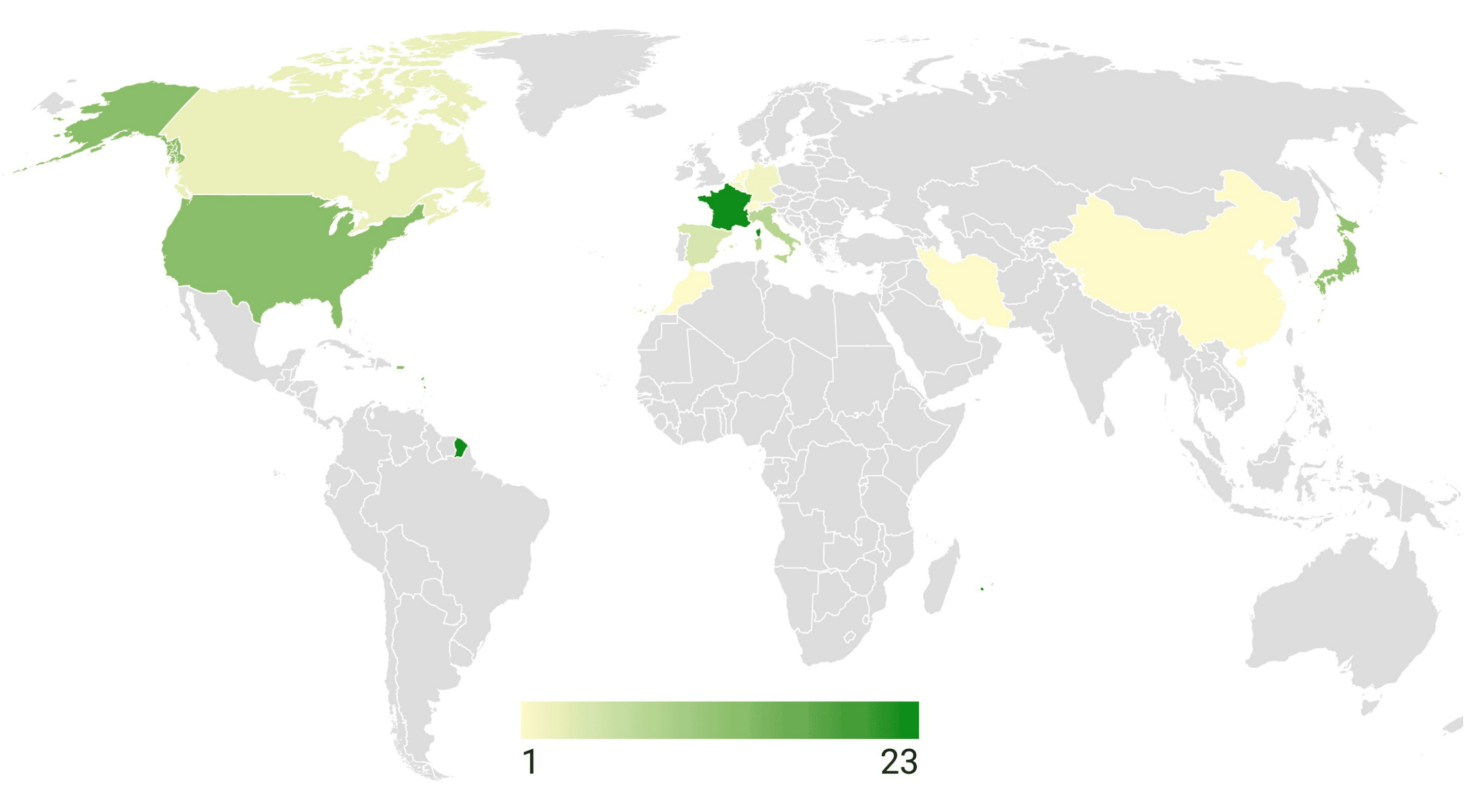
Global distribution of corresponding author affiliation by country

The global distribution of the studies revealed significant concentration in Western Europe and North America (Figure 2). Specifically, 58 studies (79%) were conducted in Western Europe and North America, 14 studies (19%) were conducted in Asia, and only one (2%) study was conducted in Africa. The top three countries were France (23 studies), the United States of America (13 studies), and Japan (12 studies). Across the 73 studies, the three commonest language error types were speech arrest (321 stimulation sites; 36.0%), anomia (213 stimulation sites; 23.9%), and dysarthria (86 stimulation sites; 9.6%). 

Distribution of Cortical Stimulation Sites Across Languages

**Figure 3:**
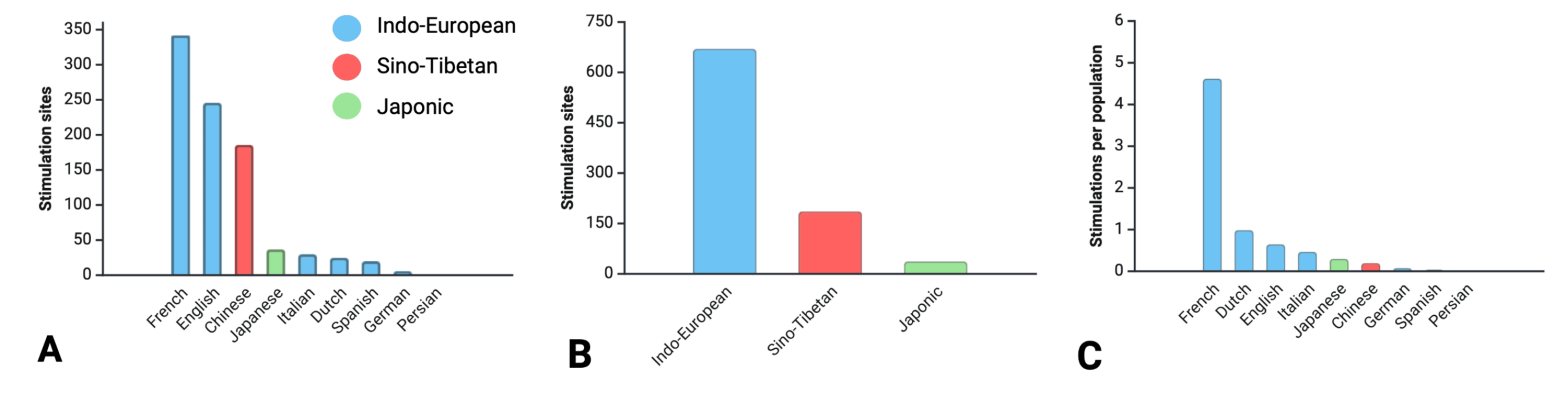
Bar charts of stimulation sites by language (A); stimulation sites by language family (B); and stimulation sites per native speaker population size (C)

From the 893 stimulation sites, we identified a total of nine languages: French, English, Chinese, Japanese, Italian, Dutch, Spanish, German, and Persian (Figure 3A). The top three mapped languages were: French (342 stimulation sites; 38.2%), English (246 stimulation sites; 27.5%), and Chinese (186 stimulation sites; 20.8%). These nine languages are spoken by 2.23 billion native speakers, which equates to 27.2% of the global population (8.2 billion humans). These nine languages were then divided into their associated language families, including: Indo-European (French, English, Italian, Dutch, Spanish, German, and Persian), Sino-Tibetan (Chinese), and Japonic (Japanese). The predominant mapped language family was Indo-European (670 stimulation sites; 75%), followed by Sino-Tibetan (186 stimulation sites; 20.8%) and Japonic (37 stimulation sites; 4.1%) (Figure 3B). We then examined the relationship between the total number of global native speakers of the language and stimulation site numbers (Table 2). Chi-squared analysis revealed a significant difference between the frequencies of the total number of global native speakers and stimulation sites (χ² = 8166.89, df = 29, p < 0.00001). We calculated a ‘stimulation site per million native speaker’ metric and identified that the top mapped languages per population size were French (4.62), Dutch (0.99), and English (0.65) (Figure 3C).

**Table 2:** Popularity of each language, with their native-speaker population and stimulation sites reported in this study

	Language	Native speakers (in millions)	Stimulation sites	Stimulation Sites per Million Speakers
1	Mandarin Chinese	941	186	0.197
2	Spanish	486	20	0.041
3	English	380	246	0.647
4	Hindi	345	0	0
5	Bengali	237	0	0
6	Portuguese	236	0	0
7	Russian	148	0	0
8	Japanese	123	37	0.300
9	Yue Chinese	86	0	0
10	Vietnamese	85	0	0
11	Turkish	84	0	0
12	Marathi	83	0	0
13	Telugu	83	0	0
14	Wu Chinese	83	0	0
15	Western Punjabi	82	0	0
16	Korean	81	0	0
17	Tamil	79	0	0
18	Egyptian Arabic	78	0	0
19	German	76	6	0.078
20	French	74	342	4.621
21	Urdu	70	0	0
22	Javanese	68	0	0
23	Italian	64	30	0.468
24	Persian	62	1	0.016
25	Gujarati	58	0	0
26	Hausa	54	0	0
27	Bhojpuri	53	0	0
28	Levantine Arabic	51	0	0
29	Southern Min	51	0	0
30	Dutch	25.3	25	0.988

Discussion

In this systematic review, we defined which languages have been mapped using DES during awake craniotomy. Of the 7000 languages spoken globally, we found only nine had been mapped using DES and described in the literature. These native languages represent just over a quarter of the global population. There was a predominance of Indo-European languages (French, English, Italian, Spanish, Dutch, German, and Persian). French was the most mapped language with DES by the number of stimulation points, but this dominance became even more pronounced when looked at as a ratio to the number of global native speakers. The large amount of data on French speakers is due to the major contributions made by Professor Hugues Duffau, a French neurosurgeon based at Montpellier. Collectively, this data highlights a bias in the literature with a predominance of Indo-European languages and a lack of representation of the native languages spoken by three-quarters of the global population. The underlying reason for this dominance of Indo-European languages is likely driven by a number of factors, including the historic concentration of global neurosurgical infrastructure in North America and Europe and a longer-established tradition of research.

Despite sharing universal features, languages exhibit remarkable variation. How this variation relates to the underlying neural architecture remains an open question. Neuroimaging has played an important role in trying to answer this question. A major study in the field examined 45 languages from 12 language families [3]. The study revealed a universal fronto-temporo-parietal language network in the human brain, predominantly left-lateralized, that supported language processing across diverse linguistic systems. Despite structural and lexical differences among the studied languages, this network exhibited consistent activation patterns, highlighting a shared neurocognitive architecture for language comprehension and production. The study had a number of limitations, including the participants being bilingual, small sample size per language (n=1-2), and a predominance of Indo-European languages (31 out of 45 languages). Neuroimaging provides important evidence on brain function. However, causal inferences on brain function cannot be made from it.

Siddiqi and colleagues defined criteria for causality in human brain mapping [4]. This included reversibility, dose response, and experimental manipulation. DES meets these criteria and is therefore a stronger experimental paradigm for causal mapping of human language function. Lu and colleagues retrospectively developed a multi-centre functional map of DES-induced speech errors by integrating four datasets comprising English, French, and Mandarin [85]. Their analysis revealed a shared fronto-temporo-parietal language network across the languages, aligning with findings from the large-scale fMRI study [3]. However, they also observed nuanced differences, such as a higher incidence of speech arrest in the posterior middle frontal gyrus in the Chinese cohort compared to the English and French cohorts, consistent with earlier research [8]. This finding highlights the value of DES in studying human language function and the importance of ensuring data from a diverse range of languages is captured from awake craniotomy surgeries. 

Currently, there is no standardized language testing paradigm for awake craniotomy. There is diversity in the test batteries used and how clinicians interpret language errors [86,87]. This is an important weakness in the field, and efforts should be made to define a core set of language tests for awake craniotomy. Importantly, these tests should be multilingual to ensure they can be used globally by awake craniotomy teams. Our group is working on developing Map-OR - a digital platform for the delivery of language tests during awake craniotomy and collaborative brain mapping [88]. Map-OR has two objectives: first, to allow the administration of standardized language tests during awake craniotomy, encompassing a variety of languages. Second, to enable the mapping of positive stimulation sites onto a digital brain atlas, with the goal of creating a prospective functional multi-language map of the human brain.

The study has several limitations. Firstly, we opted to utilise a previously published systematic review [9]. This was done as the inclusion criteria were the same as our requirements, and the review was the most comprehensive assessment of the language mapping literature to date. However, it did mean we did not conduct a fresh assessment of the literature with our own quality checks. We attempted to address this by performing a thorough review of the data quality. Additionally, the data from the original review only extended to 2020, meaning we have likely missed more recent studies that could have contributed additional stimulation data. We also found that there were 27 studies with a mixed language population. These studies were predominantly by Hugues Duffau (n=22; 81%), meaning we likely under-estimated the number of French stimulation sites. In conjunction, we did not analyse neuro-anatomical differences between the languages. This limits any conclusions about differences in the neural architecture of different languages. We plan future analyses, including novel data collected using map-OR, to answer these questions.

## Conclusions

This analysis highlights a predominance of Indo-European languages in the DES awake craniotomy literature. This work supports the need for developing a global research consortium of awake craniotomy teams to collectively pool stimulation data from a diverse range of languages. To do this, the consortium would agree on a standardized multi-language testing paradigm and then collectively pool the neuro-anatomical location of their positive stimulation sites. We are developing a digital platform (map-OR) with the objective of creating a multi-language functional map of the human brain. This endeavour would not only advance cognitive neuroscience but also enhance neurosurgical planning for a globally diverse patient population.
